# Effect of 2D and 3D ECM and Biomechanical Cues on Human iPSC‐Derived Liver Progenitor Cell Differentiation

**DOI:** 10.1002/adhm.202501370

**Published:** 2025-09-29

**Authors:** Ishita Jain, Brock Grenci, Hyeon Ryoo, Yang Yuan, Salman R. Khetani, Gregory H. Underhill

**Affiliations:** ^1^ Department of Bioengineering University of Illinois at Urbana‐Champaign Urbana IL 61820 USA; ^2^ Department of Biomedical Engineering University of Illinois Chicago Chicago IL 60612 USA

**Keywords:** 3D geometry, biomechanics, extracellular matrix cues, iPSC differentiation, liver differentiation

## Abstract

The differentiation of human liver tissue from induced pluripotent stem cells presents a powerful platform for drug screening and disease modeling. While 3D organoid systems effectively recapitulate tissue development, systematic investigation of microenvironmental parameters remains challenging. Here, complementary approaches are developed utilizing 2D cellular microarrays and 3D polyethylene glycol(PEG)‐based microwells to examine human stem cell‐derived hepatoblast differentiation. High‐throughput microarray platform enables systematic evaluation of 60 distinct microenvironmental conditions, combining 15 ECM compositions with 4 growth factor treatments. Position‐dependent expression of hepatocytic and cholangiocytic markers is observed including HNF4a, SOX9, and CK19, with differentiation patterns varying substantially across ECM and growth factor conditions. Based on these findings, specific ECM combinations are selected – Collagen 1, Fibronectin, and their combination – for integration into a modular 3D PEG hydrogel system. This 3D platform provides independent control over microtissue geometry and matrix composition, enabling investigation of spatial organization in hepatic differentiation. Through this integrated approach combining high‐throughput screening and defined 3D culture, a framework is established for dissecting the microenvironmental regulation of human liver development.

## Graphical abstract

An engineered microtissue model is developed and optimized to investigate human liver development. Extracellular matrix composition and mechanical forces are tuned using high‐throughput assays. Human induced pluripotent stem cells serve as the cellular source, enabling the observation of de novo patterned differentiation.

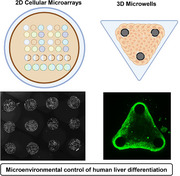

## 1 Introduction

The liver parenchyma develops through fate specification of bipotential hepatoblasts toward either hepatocytic or cholangiocytic lineages. While hepatocytes maintain metabolic homeostasis as the primary functional unit of the liver, cholangiocytes form the epithelial lining of the biliary system. The spatial organization and differentiation of these distinct cell populations are regulated by specific combinations of microenvironmental signals. Wnt signaling drives hepatic specification and establishes metabolic zonation, whereas TGFβ signaling gradients direct cholangiocyte differentiation and restrict biliary development to periportal regions.^[^ ^1^, ^2^, ^3^ ^]^ Additionally, cholangiocytic differentiation is regulated by both Notch signaling and specific Extracellular Matrix (ECM)‐integrin interactions. Notch signaling is necessary for mature bile duct formation, while integrin binding to ECM proteins like laminin can control biliary differentiation and polarization.^[^ ^4^, ^5^, ^6^, ^7^ ^]^ Pluripotent stem cells are a main source of human hepatoblast‐like cells that are studied in vitro. We have previously reported the mechanisms by which liver progenitor differentiation is simultaneously regulated by these pathways, illustrating cooperative interactions between the TGFβ and Notch pathways, which are further influenced by ECM proteins and stiffness.^[^ ^8^, ^9^ ^]^ These 2D cultures have different cell‐cell and cell‐ECM interactions compared to 3D cultures and do not fully recapitulate physiological signals such as integrin ligation or nutrient and oxygen gradients.

3D liver organoids allow for improved modeling of tissue development and function in vitro.^[^ ^10^, ^11^ ^]^ They have a wide range of cell sources and complexity with pluripotent stem cell‐derived organoids being a popular and quickly growing area of study. Stem cell‐derived organoids can self‐organize, mimic early stages of liver zonation, and include both parenchymal and non‐parenchymal cells.^[^ ^12^, ^13^, ^14^ ^]^ These cultures however, do not completely recapitulate liver function and are lacking microenvironmental cues that can impact organoid differentiation and development. Sorrentino et al. have shown the impact that substrate stiffness has on liver organoid development, demonstrating the need for well‐defined 3D organoid systems with modular microenvironmental components such as mechanical cues and ECM components.^[^ ^15^, ^16^ ^]^

Human‐derived induced pluripotent Stem Cells (hiPSCs) provide a unique opportunity to develop human liver tissue models for drug testing and patient‐based disease modelling due to the scarcity of primary liver tissue.^[^ ^17^, ^18^, ^19^ ^]^ Specific differentiation protocols using growth factor cocktails have been developed that can be implemented to obtain any lineage‐specific cells.^[^ ^20^ ^]^ hiPSC‐derived lineage‐specific cells can then be utilized for the development of various engineered tissue models. Hepatocytic differentiation from stem cells is controlled by modulating the TGFβ, Wnt, and MAPK pathways through the use of growth factors and small‐molecule drugs.^[^ ^21^, ^22^, ^23^ ^]^ These protocols allow for efficient differentiation of stem cells to definitive endoderm, hepatic progenitor cells, and finally immature hepatocytes, but they are not able to recapitulate the same level of function as mature primary hepatocytes.^[^ ^24^, ^25^, ^26^ ^]^ While these protocols try to mimic embryogenesis, they lack the same relevant microenvironmental cues that are missing in 3D organoid models.

We have previously reported how micropatterned 3D liver microtissues with defined shape and size impart unique mechanical cues to the 3D microtissue, resulting in patterned differentiation of liver progenitor cells.^[^ ^27^ ^]^ Similarly, there are other reports evaluating the effect of 3D geometry cues on tissue development, especially in the context of intestinal organoids.^[^ ^28^ ^]^ Biomaterials such as polyethylene glycol (PEG), hyaluronic acid, and gelatin have been used extensively in 3D culture systems to provide modular biomechanical and biochemical properties for modelling tissue development and diseases.^[^ ^29^, ^30^ ^]^ However, these systems lack the controlled mechanical forces imparted by systems with enhanced geometry cues, leading to variability in results obtained and a lack of multicellular tissue patterning. A combined 3D multicellular culture system guided by specific mechanical cues via the geometry of the organoid and biochemical cues via the ECM composition would emulate the in vivo microenvironment and result in more mature tissue development and function. However, hiPSCs possess some challenges in their application for the development of 3D microtissue models. Since the differentiation is performed from the pluripotent state to a specific lineage for each biological experiment, an inherent variability due to the differences in differentiation efficiency is added.^[^ ^31^ ^]^ There is a need for controlled in vitro systems with modular cues to direct intricate multicellular tissue patterning and cellular phenotype.

In this study, we investigated microenvironmental regulation of hiPSC‐derived hepatoblast differentiation using complementary 2D and 3D platforms. A high‐throughput cellular microarray system provided systematic evaluation of 60 distinct conditions, combining 15 ECM compositions with 4 growth factor treatments. Quantitative fluorescence imaging revealed spatially‐organized expression patterns of hepatocytic and cholangiocytic markers HNF4a, SOX9, and CK19, with differentiation outcomes varying substantially based on both geometry and biochemical signals. In parallel, a modular 3D PEG‐based culture system enabled independent control of microtissue geometry and matrix composition. Based on the microarray screening results, specific ECM combinations – Collagen 1, Fibronectin, and Collagen 1/Fibronectin – were selected for 3D studies due to their demonstrated effects on lineage specification and spatial patterning. The integration of high‐throughput 2D screening with defined 3D culture environments established a framework for dissecting the combinatorial effects of mechanical and biochemical cues on human hepatic development.

## 2 Results

### 2.1 Evaluation of 60 Microenvironmental Conditions for Studying Early hiHepatoblast Differentiation

**Figure 1:**
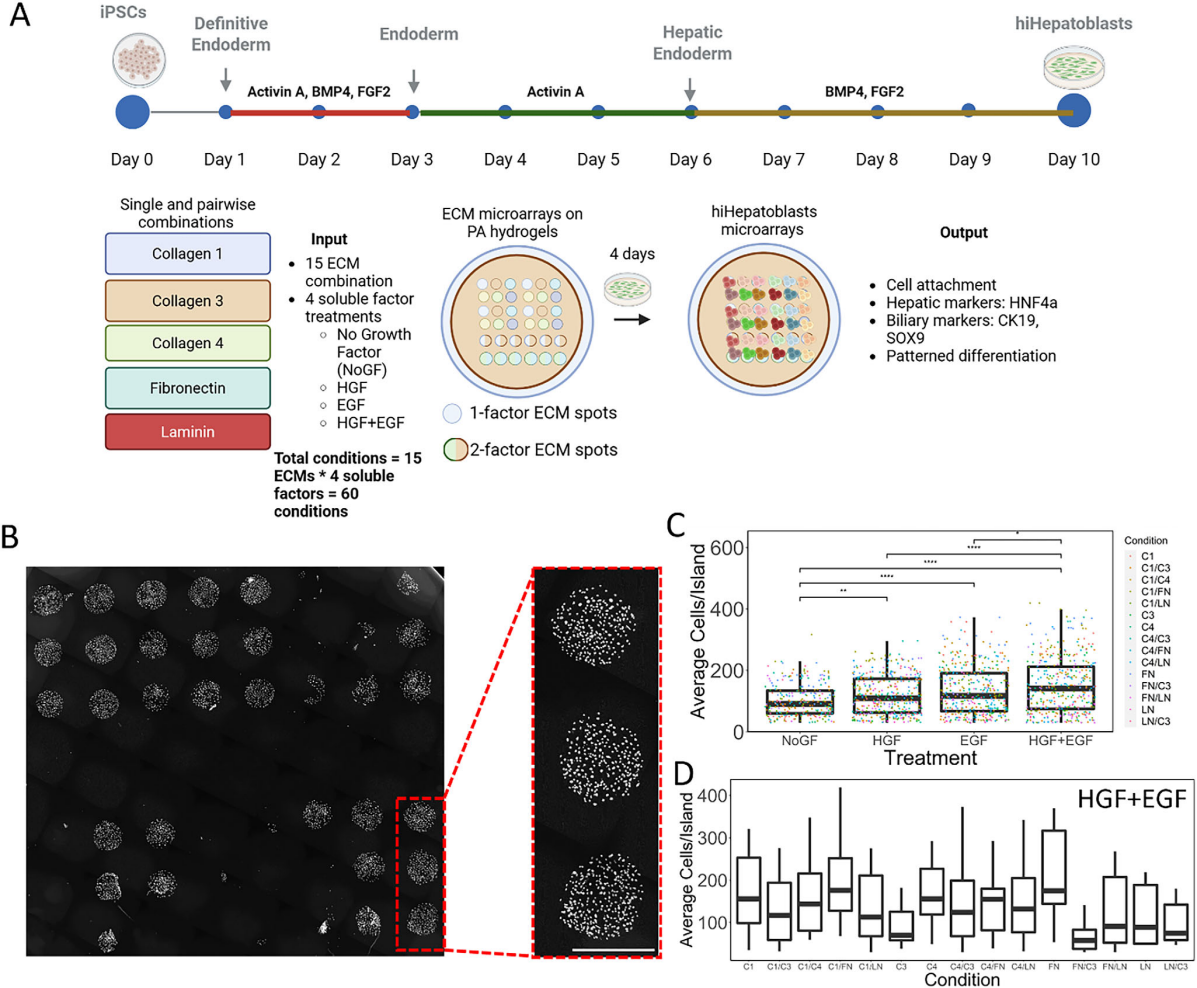
High Throughput Investigation of hiHepatoblast differentiation and cell attachment quantification as a function of 45 ECM combinations. A) Schematic of the design of a throughput microarray experiment. B) Representative image of hiHepatoblacts cultured on 15 ECM combinations on one 12 mm coverslip. Scale Bar: 600 microns. C) Quantification of average cells per island for each independent microarray island as a function of growth factor treatments. D) Quantification of average cells/island as a function of ECM combination, specifically for HGF+EGF growth factor treatment. N ≥ 3 biological replicates and 15 technical replicates. Boxplots: “^*^”: *p*‐value <0.05; “^**^”: *p*‐value <0.01; “^****^”: *p*‐value <0.0001, calculated using the Wilcox test in R, n ≥ 3 biological replicates (independent experiments) and n ≥ 10 technical replicates (individual islands).

hiPSCs (Human Induced Pluripotent Stem Cells) were differentiated to hepatoblasts (hiHepatoblasts) on Geltrex‐coated tissue‐culture plastic using the 10‐day protocol from Mallanna and Duncan.^[^ ^32^ ^]^ Briefly, hiPSCs were differentiated to the endoderm stage during Days 1–2 using Activin A, BMP4, FGF2, and B27 minus insulin, and further specified toward hepatic endoderm during Days 3–5 using Activin A and B27 minus insulin. Hepatic endoderm cells were then cultured in BMP4, FGF2, and B27 during Days 6–10 to reach the hiHepatoblast stage (**Figure** **1**a). Using these hiPSC‐derived hiHepatoblasts, we aimed to study the effect of microenvironmental components such as the ECM and growth factors on early hepatocytic and cholangiocytic fate decisions. We utilized the high‐throughput microarray technology^[^ ^33^ ^]^ to study single and pairwise combinations of 5 basal lamina proteins – Collagen 1, Collagen 3, Collagen 4, Fibronectin, and Laminin as they have previously been shown to mediate hepatic progenitor cell differentiation.^[^ ^9^ ^]^ These ECM components undergo dynamic remodeling during liver development and disease progression.^[^ ^34^, ^35^, ^36^, ^37^ ^]^ Collagen 4 and Laminin constitute the primary components of mature bile duct basement membranes in adult liver, and Fibronectin is abundant during early hepatogenesis and has been shown to support hepatoblast migration.^[^ ^36^, ^38^ ^]^ Collagen 1 deposition has been shown to increase during later stages of development and has been suggested to enhance hepatocyte maturation.^[^ ^35^, ^36^ ^]^ Further, we wanted to elucidate the combinatorial effect of these ECMs with growth factor treatments – HGF, EGF, and HGF+EGF. HGF has been shown to play an important role in lineage specification of immature hepatocytes,^[^ ^39^ ^]^ whereas EGF has been shown to influence Notch signaling in cholangiocytic differentiation.^[^ ^40^ ^]^ Combined treatment of HGF+EGF has been used to obtain mature functional hepatic organoids with multi‐lineage differentiation.^[^ ^41^, ^42^, ^43^ ^]^ Figure 1a demonstrates the overall experimental design to study the effect of 60 microenvironmental combinations on hepatic differentiation of hiPSC‐derived hepatoblasts (hiHepatoblasts). A representative image of the cellular microarrays is shown in Figure 1b, where hiHepatoblasts are cultured for 4 days on the microarrays prior to fixation. Each circular spot (600um in diameter) is a unique ECM combination contact‐printed on a polyacrylamide hydrogel, referred to as the island. A differential number of hiHepatoblasts/island was observed for a specific ECM combination and growth factor treatment. Furthermore, significantly higher numbers of cells were found on every ECM island with HGF, EGF, and HGF+EGF treatment compared to the no growth factor (NoGF) control (117, 121, and 145 cells per island vs 101 cells perisland; *p*‐value < 0.01) (Figure 1c). Additionally, the highest number of cells per island was observed with HGF+EGF treatment, indicating better cell attachment and survival (Figure 1c). The variation in hiHepatoblast cell number as a function of the underlying ECM combination with the HGF+EGF treatment is demonstrated in Figure 1d. Collagen 1 (170 cells per island), Fibronectin (203 cells per island), and Collagen 1+Fibronectin (204 cells per island) were some of the ECM conditions exhibiting the highest cell numbers compared to Collagen 3 (106 cells per island) and Fibronectin + Collagen 3 (85 cells perisland). Overall, the high‐throughput microarray technology enabled evaluation of hiHepatoblast attachment and survival while highlighting the synergistic effect that varying cell‐soluble factor and cell‐ECM interactions have on differentiation in vitro.

### 2.2 Quantification of Biliary Differentiation of hiHepatoblasts on the High‐Throughput Microenvironmental Conditions

**Figure 2:**
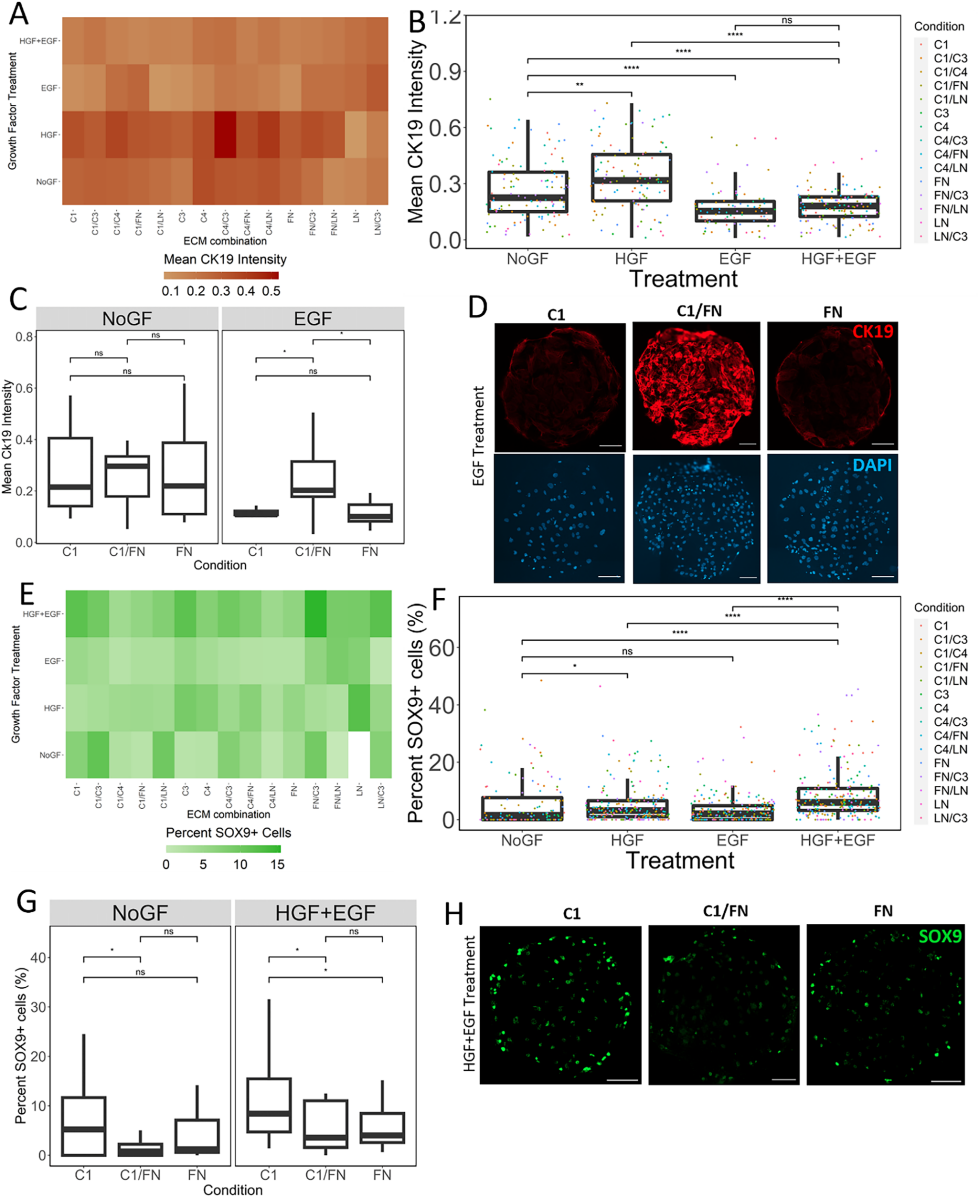
Quantification of cholangiocytic lineage markers as a function of growth factors and ECM combinations. A) Heatmap of average CK19 fluorescent intensity of hiHepatoblasts as a function of combinatorial ECMs and growth factor treatments. B) Box plot of mean CK19 intensity of hiHepatoblasts as a function of growth factor treatments. C) Box plot of mean CK19 intensity as a function of ECMs Collagen 1(C1), Collagen 1 and Fibronectin (C1/FN) & Fibronectin (FN) for NoGF control and EGF growth factor treatment. D) Representative fluorescent images for hiHepatoblast islands on different ECMs with EGF growth factor treatment. Red: CK19, Blue: DAPI. Scale Bar 100 microns. E) Heatmap of average %SOX9+ hiHepatoblasts as a function of combinatorial ECMs and growth factor treatments. F) Box plot of mean %SOX9+ hiHepatoblasts as a function of growth factor treatments. G) Box plot of mean %SOX9+ hiHepatoblasts as a function of ECMs Collagen 1(C1), Collagen 1 and Fibronectin (C1/FN) & Fibronectin (FN) for NoGF control and HGF+EGF growth factor treatment. H) Representative fluorescent images for hiHepatoblast islands on different ECMs with EGF growth factor treatment. Green: SOX9. Scale Bar 100 microns. Boxplots: “^*^”: *p*‐value <0.05; “^**^”: *p*‐value <0.01; “^****^”: *p*‐value <0.0001, calculated using the Wilcox test in R, n ≥ 3 biological replicates (independent experiments) and n ≥ 10 technical replicates (individual islands).

Focusing on the biliary lineage, we quantified expression of both a functional marker of biliary lineage, CK19, and a biliary transcription factor, SOX9. Significant variation in CK19 expression as a function of both ECM and growth factor treatment was observed (**Figure** **2**a). Specifically, HGF treatment resulted in a 27% increase in CK19, while the EGF and HGF+EGF treatments resulted in a 33% and 31% decrease, respectively, in overall CK19 intensity expression compared to the no growth factor (NoGF) condition (*p* < 0.05) (Figure 2b). EGF has been shown to promote biliary cell differentiation and proliferation, contrary to what we see with CK19 expression.^[^ ^44^, ^45^ ^]^ While EGF decreased overall CK19 expression, certain ECM conditions upregulated CK19 regardless of growth factor treatment (Figure 2c). Specifically, both Collagen 1 and Fibronectin alone downregulated CK19 with EGF treatment in hiHepatoblasts, but Collagen 1 and Fibronectin combined resulted in a 136% increase in CK19 intensity (*p*‐value < 0.05). This demonstrates that while growth factor treatments shift overall CK19 expression, cooperative effects between ECMs and growth factors can further modulate biliary expression. Representative images of CK19 staining on varying ECMs with EGF treatment are shown in Figure 2d. Next, SOX9 expression was evaluated for the same unique ECM and growth factor combinations (Figure 2e). Specifically, it was noted that HGF+EGF growth factor treatment significantly increased the percent of SOX9 positive cells compared to HGF treatment and the NoGF control (6% SOX9+ cells versus 3.1% and 1% SOX9+ cells, respectively, *p*‐value < 0.01) (Figure 2f). Similar to CK19, SOX9 expression was further affected by certain combinations of ECMs and growth factor treatments. While HGF+EGF promoted SOX9 expression overall, Collagen 1 pushed a 2‐fold higher percentage of the cells toward a biliary lineage compared to Fibronectin and Collagen 1 and Fibronectin combined (8.4% SOX+ cells versus 4% and 3.5% SOX+ cells respectively, *p*‐value < 0.05) (Figure 2g). Representative islands for different ECM conditions with the HGF+EGF treatment are shown in Figure 2h. Overall, both CK19 and SOX9 expression are influenced strongly by growth factor treatment and can be further regulated by ECM in combination with these growth factors. This synergistic interaction between ECM composition and growth factor signaling represents a potential regulatory mechanism for biliary differentiation, as evidenced by the dramatic CK19 response when Collagen 1 and Fibronectin are combined with EGF treatment. Such cooperative regulation may help explain the complex spatial patterning of biliary structures observed during liver development, where localized ECM deposition coincides with growth factor gradients to establish precise ductal architecture.

### 2.3 Levels of Hepatic Transcription Factor HNF4a Varied Extensively with the Underlying ECM Combination

**Figure 3:**
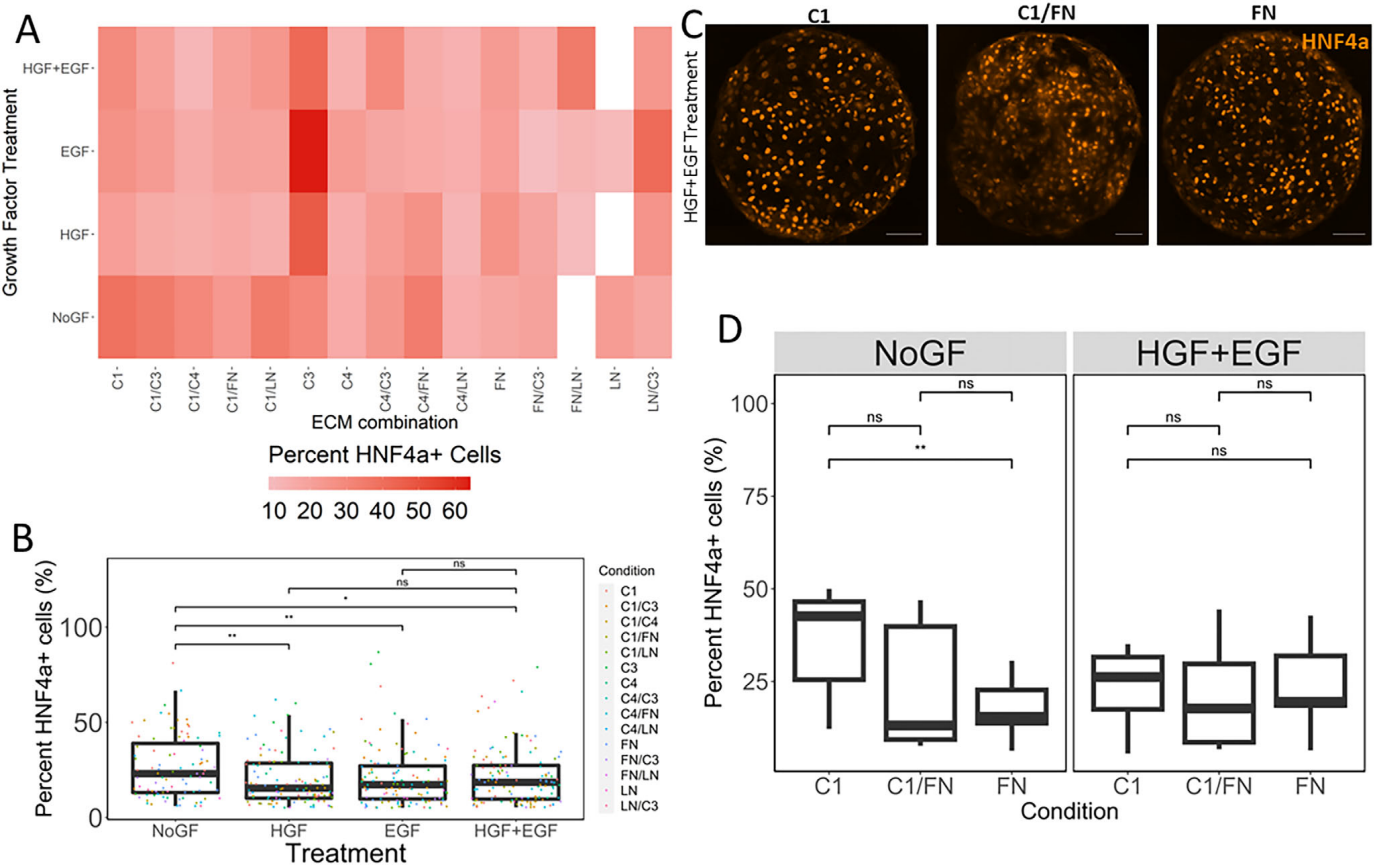
Quantification of hepatic lineage markers as a function of growth factors and ECM combinations. A) Heatmap of average %HNF4a+ hiHepatoblasts as a function of combinatorial ECMs and growth factor treatments. B) Box plot of mean %HNF4a+ hiHepatoblasts as a function of growth factor treatments. C) Representative fluorescent images for hiHepatoblast islands on different ECMs with HGF+EGF growth factor treatment. Red: HNF4a. Scale Bar 100 microns. D) Box plot of mean %HNF4a+ hiHepatoblasts as a function of ECMs Collagen 1(C1), Collagen 1 and Fibronectin (C1/FN) & Fibronectin (FN) for NoGF control and HGF+EGF growth factor treatmentRed: Boxplots: “^*^”: *p*‐value <0.05; “^**^”: *p*‐value <0.01; calculated using Wilcox test in R, n ≥ 3 biological replicates (independent experiments) and *n* ≥ 10 technical replicates (individual islands).

Differentiation toward a hepatic lineage was quantified using a fluorescent stain against a hepatocytic transcription factor, HNF4a. **Figure** **3**a depicts the HNF4a expression of differentiated hiHepatoblasts as a function of the combinatorial ECM and growth factor treatments and demonstrates the overall variability in cellular phenotype caused by these different microenvironmental conditions. All growth factor treatments, HGF, EGF, and HGF+EGF led to a significant decrease in the % HNF4a+ cells compared to the NoGF control (15%, 17%, and 18% HNF4a+ cells versus 23% HNF4a+ cells, *p*‐value < 0.05) (Figure 3b). The NoGF control was enough to drive hepatic differentiation independent of the growth factor treatments on the microarrays, showing that the variable HNF4a expression shown in Figure 3a is heavily influenced by ECM combinations.

ECM combinations like Collagen 1 (42% HNF4a+ cells) and Collagen 4+Fibronectin (34% HNF4a+ cells) had the highest percent of HNF4a positive cells for the NoGF control. However, ECMs combinations like Fibronectin (only) and Collagen 3(only) led to higher percentages of HNF4a positive cells with growth factor treatments. While ECM had a strong influence on HNF4a expression, growth factor treatments had the ability to silence certain ECM effects. Figure 3c depicts representative images of Collagen 1, Fibronectin, and Collagen 1+Fibronectin islands with HGF+EGF treatment. While Collagen 1 islands had twice as many HNF4a+ cells compared to Fibronectin islands in the NoGF treatment, there was no significant difference between HNF4a+ cells on Collagen 1 or Fibronectin islands when treated with HGF + EGF (Figure 3d). This shows that while HNF4a expression is driven strongly by ECM composition, those effects can be modulated by growth factor treatment. In contrast, biliary marker expression was more heavily influenced by growth factor treatments and modulated by ECM.

### 2.4 Spatially Organized Differentiation of hiHepatblasts Observed as Function of Growth Factors

**Figure 4:**
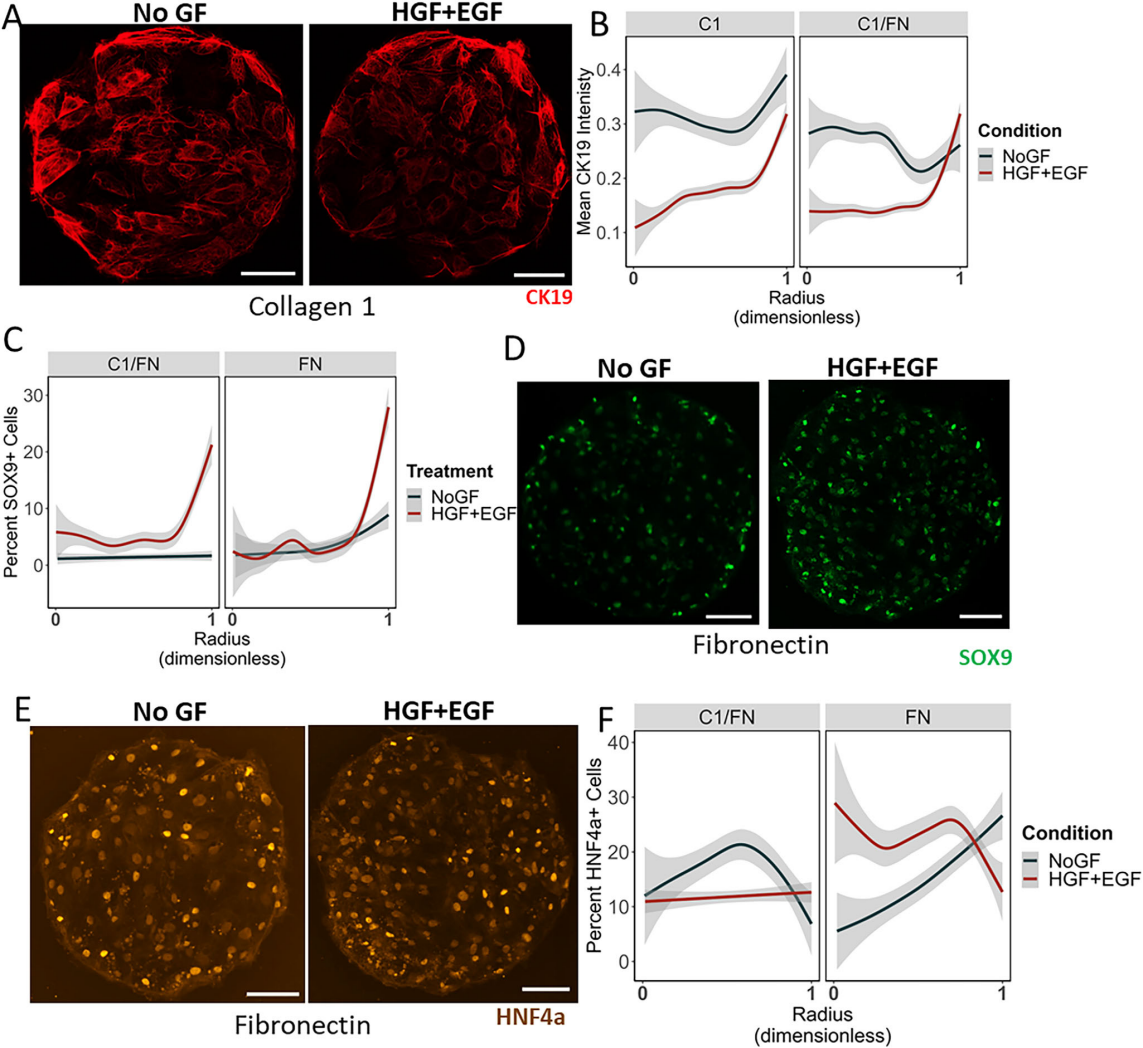
Quantification of patterned bipotential differentiation of hiHepatoblast islands as a function of ECM and Growth Factors. A,D,E) Representative fluorescent images for CK19, SOX9, and HNF4a staining on hiHepatoblast islands on Collagen 1 and Fibronectin ECMs, respectively with NoGF and HGF+EGF growth factor treatment. Red: CK19; Green: SOX9; Brown: HNF4a. Scale Bar 150 microns. B,C,F) Quantification of average CK19 Intensity, %SOX9+ cells, and %HNF4a+ cells as a function of the radius on the circular island for different ECMs and growth factor conditions. 0 is the center, 1 is the edge. Gray: 95% confidence interval.

We have previously reported patterned bipotential differentiation of mouse hepatic progenitors on these circular microarrays due to the biomechanical gradients as a result of the geometry.^[^ ^46^ ^]^ Higher biliary differentiation was observed on the boundary of the circular microarrays, while higher hepatic differentiation was seen on the interior. We had also discovered crosstalk of underlying signaling pathways, specifically Notch signaling and E‐Cadherin‐mediated cell‐cell crosstalk that influenced cell fate across the islands.^[^ ^47^ ^]^ We wanted to evaluate if a similar differentiation pattern was observed using hiHepatoblasts. Patterned expression for all stains was quantified as a function of island radius, with 0 being the center and 1 being the boundary of the cellular microarray. **Figure** **4**a demonstrates how the location of CK19‐expressing cells varies depending on ECM and growth factor treatment. A spatial CK19 expression pattern was observed among the differentiated hiHepatoblasts as a function of island geometry, specifically in the HGF+EGF condition. Low CK19 expression was found on the interior of the circular microarray, and exponentially higher CK19 expression on the boundary was quantified (Figure 4b). The NoGF condition did not show the same level of CK19 upregulation in the boundary. Additionally, we further evaluated the biliary pattern on the microarrays by quantifying the percentage of SOX9+ cells. A pattern was observed for many ECM conditions with HGF+EGF treatment. Specifically, low amounts of SOX9+ cells were observed across the interior, and an exponential increase was observed right at the boundary of the circular microarray (Figure 4c,d). Comparatively, the NoGF treatment did not show the same exponential increase near the edge of the islands for the Fibronectin and Collagen 1+Fibronectin islands. In conclusion, both CK19 and SOX9 expression had a spatial pattern on the microarrays, specifically when treated with HGF+EGF.

HNF4a expression was also quantified as a function of a cell's radial position, and representative images of HNF4a expression on Fibronectin islands are shown in Figure 4e. For the NoGF treatment, most ECM conditions exhibited a contradictory pattern to what was observed in our previous investigations, where either no spatial pattern or a higher percentage of HNF4a+ cells on the boundary was observed. However, with HGF+EGF treatment, a similar pattern to our previous studies was observed. Specifically, single‐factor ECM islands like Collagen 1 and Fibronectin exhibited higher HNF4a+ cells across the interior of the cellular microarray, and the number of HNF4a+ cells decreased exponentially at the edge (Figure 4f). Overall, HNF4a expression was found to be primarily dependent on the underlying ECM combination, whereas the localization on the circular island was found to be primarily influenced by the growth factor treatment. It was noted that both the hepatocytic and biliary markers showed a spatial expression pattern specifically with the HGF+EGF treatment. Moreover, Collagen 1 and Fibronectin single‐factor combinations were the ECMs that showed the strongest spatial localization of differentiation markers. Based on our previous delineation of the differentiation pattern, we know that the pattern is a result of coordinated cell‐cell forces and communication. Hence, our results with the HGF+EGF treatment with Collagen 1 and Fibronectin signified the formation of better cell‐cell contacts and coordinated bifurcation of lineage fate.

### 2.5 Principal Component Analysis of 60 Microenvironmental Conditions and Its Effect on Differentiation of hiHepatoblasts

**Figure 5:**
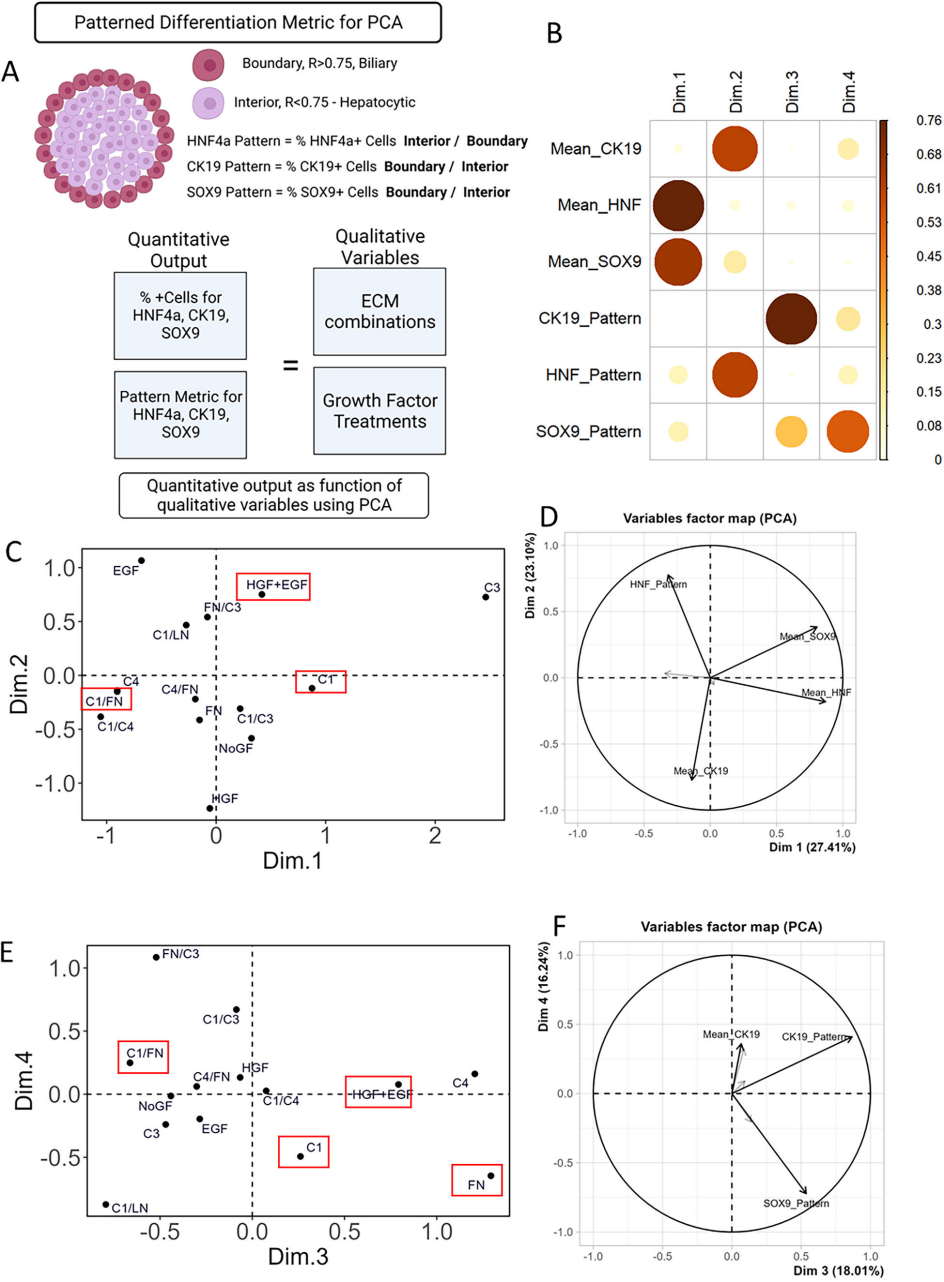
Principal component analysis (PCA) of high‐throughput data from the 2D microarray studies. A) Schematic of calculating the patterned differentiation metric for the three differentiation markers: CK19, HNF4a, and CK19 with the overall equation for the PCA. B) Contribution of each metric (Mean CK19, HNF4a, and SOX9 expression and pattern values) on the first four principal components. C,E) Position of each growth factor and ECM on the principal component planes 1 and 2, respectively. D,F) Directional position of each quantitative metric (Mean CK19, HNF4a, and SOX9 expression and pattern values) on the principal component planes. Analysis done in R, using factomineR and factorextra packages.

Principal component analysis is a quantitative method to reduce dimensions in a dataset by finding new dimensions with the most variability. This facilitates visualization of high‐dimensional data in a digestible format and enables unique insights. We aimed to analyze the expression and spatial pattern of the 3 bipotential differentiation markers (HNF4a, SOX9, and CK19) as a function of the underlying ECM conditions and growth factor treatment. The spatial expression pattern was included in the PCA analysis by incorporating a “spatial pattern coefficient” for all three differentiation markers (**Figure** **5**a). Based on our previous reports, we expected hepatocytic differentiation on the interior of the circular island, so the spatial coefficient of HNF4a was calculated as %HNF4a+ cells in the interior versus the boundary. On the contrary, since we expected biliary differentiation on the boundary of circular islands, the spatial pattern coefficient was calculated as %CK19+ cells and %SOX9+ cells on the boundary versus the interior. Overall, on principal component plane 1, 50% variability in the full dataset was explained, with mean HNF4a and SOX9 expression correlating the best with Dimension 1 (Figure 5c,d). Notably, the unique ECM combinations varied along Dimension 1, signifying the importance of ECM composition in directing the expression of HNF4a and SOX9. Using the variable factor map, we also observed directionality in SOX9 and HNF4a variability, which correlated with each other. The C1 ECM combination positioned on the positive side of Dimension 1 represented high SOX9 and HNF4a expression compared to C1+FN ECM combination lying on the negative side of Dimension 1. Furthermore, expression of CK19 and HNF4a spatial pattern correlated well with Dimension 2 (Figure 5b), with growth factor treatments distributed across Dimension 2 (Figure 5c). Since HNF4a spatial pattern and CK19 expression values corresponded to opposite directions on Dimension 2 (Figure 5d), growth factor treatments HGF+EGF and EGF positioned on positive Dimension 2 led to higher HNF4a patterning compared to HGF and NoGF conditions, which lay on the negative side of Dimension 2. Since only 50% of the whole variability in data was explained by Dimensions 1 and 2, it was necessary to analyze Dimensions 3 and 4 (Figure 5b,e,f). Overall, the CK19 pattern correlated with Dimension 3, with the growth factor treatments varying across this dimension. Lastly, the SOX9 pattern correlated with Dimension 4, with the unique ECM combinations directing the variability along this dimension. In summary, the ECM combination was found to be the most significant factor in determining the expression of the differentiation markers, while growth factor treatment guided the pattern formation of the differentiation markers.

### 2.6 Development of 3D hiHepatoblast Microtissues with Defined ECM Combination, Shape, and Size

**Figure 6:**
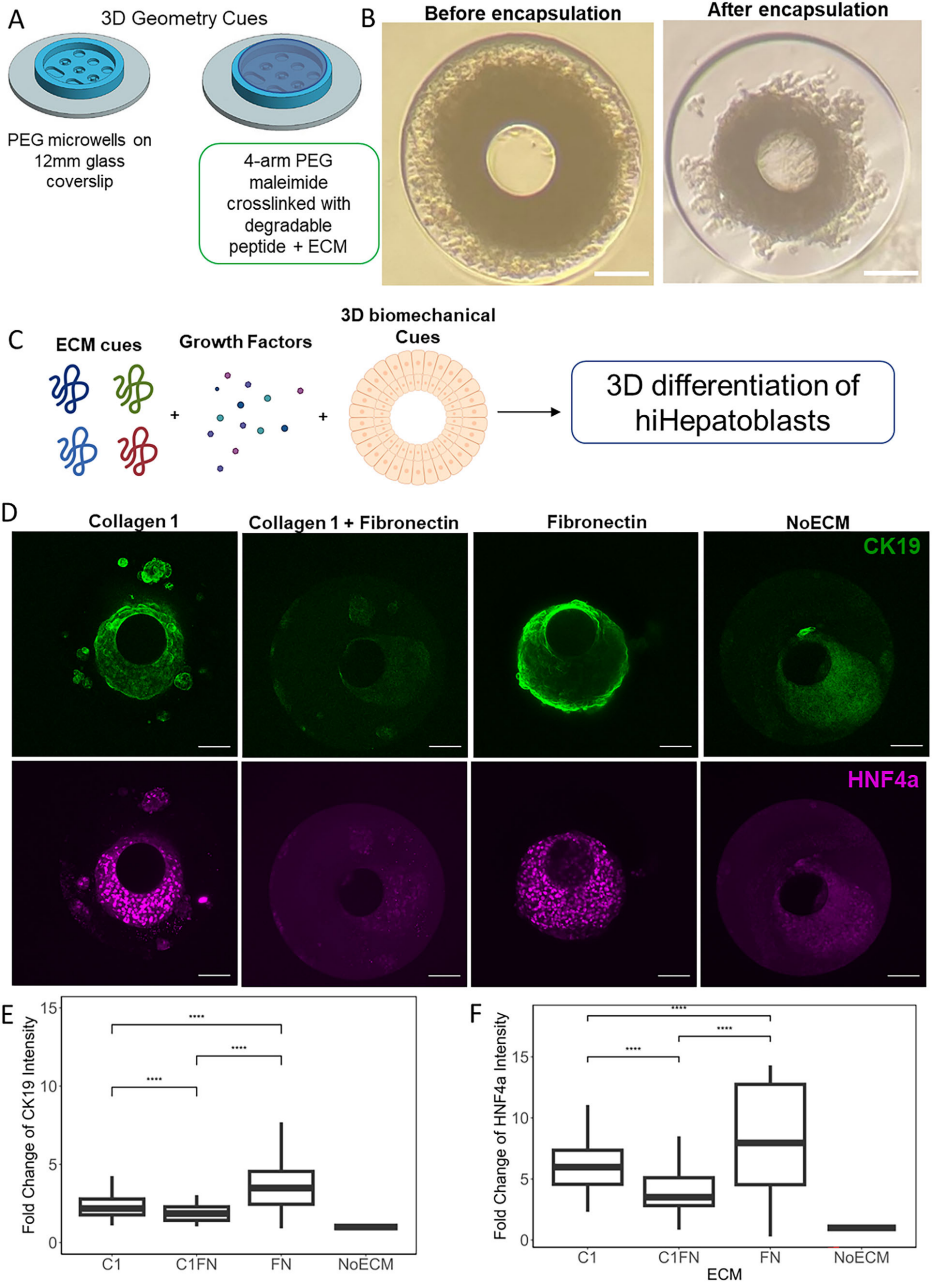
Design of 3D modular hydrogel system to study 3D hiHepatoblast microtissues with defined ECM and geometry cues. A) Schematic of the design of the 3D modular hydrogel system. B) Representative brightfield image of 3D microtissues before and after encapsulation with PEG‐ECM hydrogel. C) Schematic of experimental design of study 3D hiHepatoblast differentiation with defined ECM, growth factor, and geometry cues. D) Representative images of hiHepatoblast microtissue with different PEG‐ECM hydrogels. Green: CK19, Purple: HNF4a. Scale Bar: 150 microns E,F) Quantification of mean fold change of CK19 and HNF4a intensity compared to NoECM control for the encapsulation hydrogel. Boxplots: “^*^”: *p*‐value <0.05; “^****^”: *p*‐value <0.0001; calculated using the Wilcox test in R, n ≥ 3 biological replicates.

A spectrum of differentiation behavior of hiHepatoblast was analyzed on 60 different microenvironmental conditions using the high‐throughput cellular microarrays. Next, we aimed to design a modular 3D system with specific ECM and biomechanical cues to direct tissue patterning and differentiation of hiHepatoblasts by utilizing insights from the high‐throughput analysis. PEG microwells with a defined 3D shape established in a previous investigation were utilized to study this further.^[^ ^27^ ^]^ The PEG microwells are 3D wells with defined shape and size made using PDMS molds. The non‐fouling nature and biomechanical properties of PEG‐acrylate enabled better resolution of shape transfer and enabled the formation of microtissues by only providing mechanical support to cells. An ECM component was added to these microwells by adding a PEG‐ECM hydrogel on top of the microwells. For these experiments, Day 10 hiHepatoblasts were cultured in the PEG microwells (**Figure** **6**a). It was noted that the hiHepatoblast microtissues required support pillars to tether for maintaining the shape of a microwell. The hiHepatoblasts were cultured in the microwells for 24 h before encapsulation of the microtissues using a degradable PEG‐ECM hydrogel. Utilizing thiol‐ene chemistry, ene bonds of PEG maleimide were crosslinked to thiol bonds in VPM, a degradable peptide. The specific ECM was added in the pre‐polymer solution before crosslinking and was entrapped in the PEG hydrogel after crosslinking. Three ECM combinations, Collagen 1 (C1), Fibronectin (FN), and Collagen 1+Fibronectin (C1FN) were chosen for further evaluation in 3D since they demonstrated the best cell attachment and patterned differentiation in the high‐throughput cellular microarray investigation. HGF+EGF growth factor combination resulted in high cell number islands, bipotential differentiation, and most importantly, spatial patterning of the hepatocytic and biliary fate marker, indicating effective bifurcation of fate. Hence, for all 3D PEG microwell studies, HGF+EGF treatment was used. The hiHepatoblasts were cultured in the encapsulated 3D ECM PEG hydrogel (Figure 6b) for various timepoints; however, they lost their shape and became tethered to the pillar after 48 h. Hence, to evaluate the effect of unique mechanical forces imparted by the shape of the microwells, hiHepatoblast microtissues were evaluated at 48 h after encapsulation. Overall, a 3D modular PEG hydrogel system was designed with controllable ECM cues, growth factor treatments, and geometrical cues (Figure 6c). Bipotential differentiation of 3D hiHepatoblasts as a function of 3D ECM cues was quantified using CK19 (biliary) and HNF4a (hepatic) stains. A significant increase of 3.5‐fold and 2.2‐fold in CK19 intensity for Fibronectin and Collagen 1 ECM combinations was observed compared to NoECM control (*p*‐value <0.05) (Figure 6d,e). Similarly, a significant increase of 8‐fold and 6‐fold in HNF4a intensity for Fibronectin and Collagen1 ECM combinations was quantified compared to NoECM control (p‐value< 0.05) (Figure 6d,f). This signified the importance of 3D ECM cues influencing bipotential differentiation of hiHepatoblasts. Additionally, a significant increase in both biliary and hepatic markers in Collagen 1 and Fibronectin conditions was observed compared to those of the ECMs together, which is what was also observed on microarray studies (Figure 6d–f). Overall, a new 3D PEG hydrogel system was designed with modular ECM properties guiding hiHepatoblast bipotential differentiation.

### 2.7 Combinatorial ECM Dependent Patterned Hepatic Differentiation in 3D hiHepatoblast Microtissues

**Figure 7:**
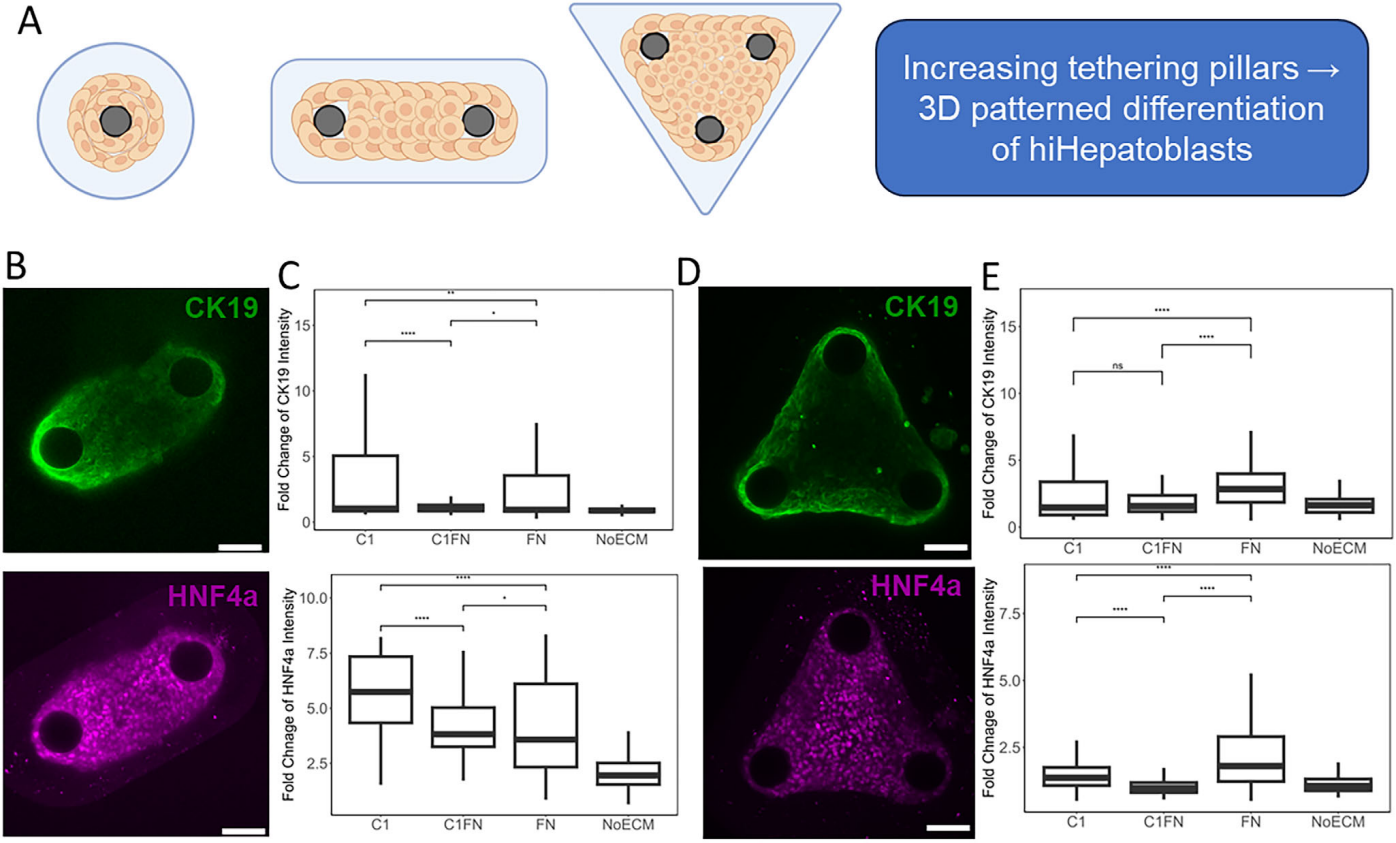
Optimization of 3D microtissues by adding tethering support pillars for defined mechanical forces. A) Schematic of the design of adding a tethering support pillar to guide different geometries of 3D microtissues. B,D) Representative images of hiHepatoblast microtissues with 2 and 3 tethering pillars, respectively, in PEG‐Fibronectin hydrogel. Green: CK19, Purple: HNF4a. Scale Bar: 150 microns C,E) Quantification of mean fold change of CK19 and HNF4a intensity compared to NoECM for 2 and 3 tethering pillars, respectively. Boxplots: “^*^”: *p*‐value <0.05; “^****^”: *p*‐value <0.0001; calculated using the Wilcox test in R, n ≥ 3 biological replicates.

Our aim was to study 3D patterned differentiation in hiHepatoblasts in the 3D microtissues as a function of geometric cues. In our single tethering pillar microtissues, slight patterning and bifurcation of CK19 and HNF4a intensity were observed, but it was inconsistent. hiHepatoblasts tended to condense extensively around the pillar or break into smaller spheres. In the efforts to optimize this and get uniform 3D shapes with mechanical gradients to drive patterned differentiation, multiple tethering pillars in different shapes and orientations were incorporated, resulting in consistent microtissues and observation of patterned differentiation. Specifically, 2 tethering pillars resulting in an elongated 3D microtissue and 3 tethering pillars in a triangular orientation were implemented (**Figure** **7**a). In the 2‐pillar microtissues, the effect of mechanical gradients due to tethering around pillars was seen on CK19 intensity, especially. Higher CK19 intensity was observed around the pillars, which was similar to our microarray reports of higher tensile forces leading to higher biliary differentiation (Figure 7b). A significantly better‐defined spatial pattern was observed with the triangular orientation of the tethering pillar, where CK19 was observed consistently on the outside of the 3D microtissues and HNF4a on the inside (Figure 7d). It was also noted that ECM effects for both these confirmations followed similar trends as seen in the single pillar 3D microtissues. Specifically, Collagen 1 and Fibronectin ECM led to significantly higher fold changes in CK19 and HNF4a intensities compared to NoECM controls (*p*‐value<0.05) (Figure 7c,e). Similar to our findings in 2D studies, the spatial patterning of bipotential differentiation and absolute intensities of differentiation can be modulated independently, even in 3D. Specifically, 3D ECM cues influenced the absolute expression of CK19 and HNF4a, whereas the 3D geometry influenced spatial patterning due to precise mechanical cues.

## 3 Discussion

In this study, we evaluated the effect of microenvironmental conditions on early differentiation of hiHepatoblasts using an integrated platform of a 2D high‐throughput cellular microarrays and a 3D microwell system. The 2D high‐throughput cellular microarray system enabled high‐throughput investigation of 60 different microenvironmental combinations consisting of 15 unique ECM combinations and 4 growth factor treatments. This enabled a detailed study of early human liver differentiation where hepatocytic and cholangiocytic lineage markers were quantified using HNF4a, SOX9, and CK19. Furthermore, patterned differentiation was also observed as a result of the geometry cues signaling a key bifurcation step in liver differentiation from the microarrays. This study also found that the degree of the patterned differentiation varied extensively as a function of ECM and growth factor. Principal Component Analysis enabled quantification of specific effects of ECM and growth factor on both mean expression and spatial pattern of bipotential differentiation markers. Simultaneously, a 3D microwell system was also developed to study the effect of defined 3D geometry and microenvironmental cues on early differentiation of hiHepatoblasts. Optimal growth factor treatment and ECM combinations for the 3D culture were chosen based on the results from the 2D cellular microarray experiments. In particular, Collagen 1, Fibronectin, and Collagen 1 + Fibronectin were chosen based on their influence on bipotential differentiation and subsequent patterning response. The 3D microwell system was developed as a modular PEG‐based culture system with independent modulation of the 3D microtissue shape and ECM composition. In conclusion, an integrated platform of 2D high‐throughput and 3D modular hydrogels was developed with the incorporation of both specific geometry and ECM cues in 3D to study human liver development.

Based on our evaluation of the 60 microenvironmental conditions on the bipotential differentiation of hiHepatoblasts, unique observations regarding bipotential differentiation were made. Both HNF4a and CK19 are makers of a progenitor state as well as differentiated hepatocytes and cholangiocytes, respectively. During development, periportal hepatoblasts express CK19, which later is exclusive to only the cholangiocytes.^[^ ^38^, ^48^ ^]^ Because of the common markers between hepatic progenitor cells and the differentiated cells, it becomes harder to categorize cells solely based on the expression profiles of a few markers. Co‐expression of multiple markers is required. For future work, further analysis of the expression of additional markers such as TBX3, CK8, and CK18, which are specifically hepatoblast markers, will be necessary. However, patterned expression of these markers on the circular microarray indicates bifurcation of fates of the hiHepatoblasts, with spatial segregation of the hepatocytic and cholangiocytic markers enabled by traction force gradients and an HGF+EGF growth factor condition. In our previous detailed evaluation of the patterned differentiation of the embryonic mouse bipotential liver progenitor cells, the patterned differentiation was found to be a result of an intricate balance between cell‐cell interaction and cell‐substrate interactions.^[^ ^47^ ^]^ This intricate balance depended highly on E‐Cadherin expression and interaction based on the number of cells on a single circular microarray. With the different growth factor conditions in the current study, HGF+EGF had the highest number of cells/island, promoting better cell‐cell interactions between cells, and moreover leading to a unified behavior of the cells on an island. It was also noted that this patterned behavior, which is a great model to study the bifurcation of fate of hiHepatoblasts, varied extensively with the underlying ECM composition.

During liver development, liver progenitor cells acquire varying roles of migration, expansion, and differentiation based on the external cues from the surrounding mesenchymal cells. This involves changes in the basement membrane composition and subsequent changes in cell‐substrate interactions. We observed high HNF4a, SOX9, and CK19 expression with Collagen 1 or Fibronectin individually; however, when both ECMs were combined, they produced lower expression of these markers despite supporting higher cell numbers per island. This striking difference is indicative of a significant phenotype change in hiHepatoblasts as a function of the underlying ECM combination. These findings align with previous studies in mouse models where ECM composition dynamically changes during liver development. In mouse embryonic liver, Collagen 1 deposition increases during the later stages of hepatogenesis and has been shown to promote hepatocyte maturation through integrin‐mediated signaling,^[^ ^34^, ^35^ ^]^ while Fibronectin is abundant in early liver development and supports hepatoblast proliferation and migration.^[^ ^36^, ^37^ ^]^ The combined effect of these ECMs in our system suggests a potential recapitulation of transitional developmental stages, where cells may be maintaining a more progenitor‐like state with higher proliferative capacity but lower differentiation marker expression. This phenomenon highlights the importance of precisely controlled ECM composition in directing liver cell fate decisions. Further, the finding that Collagen 4 and Laminin combinations did not strongly promote biliary differentiation contrasts with their role in mature bile duct basement membranes in adult liver.^[^ ^49^ ^]^ This apparent discrepancy may reflect the developmental stage of the hiHepatoblasts studied here, which likely represent early progenitor cells rather than mature cholangiocytes that typically interact with adult biliary ECM compositions. The unexpected decrease in CK19 expression with EGF treatment, contrary to its previously suggested role in promoting biliary differentiation,^[^ ^44^, ^45^ ^]^ also highlights the complex context‐dependent nature of growth factor signaling in liver development. This finding is consistent with reports demonstrating that the effects of EGF on cholangiocyte markers can be altered by substrate stiffness and ECM composition.^[^ ^15^, ^49^ ^]^ The differential expression of CK19 and SOX9 to growth factor treatments also emphasizes that these biliary markers, while often co‐expressed, are potentially regulated through distinct signaling pathways that exhibit varying responses to microenvironmental context.^[^ ^4^, ^6^, ^40^ ^]^

This study also presents a hybrid approach of utilizing both a 2D and 3D system to develop better human liver in vitro models to study liver development and diseases. In our 3D modular PEG hydrogel system, we demonstrated the capability of imparting specific geometrical cues via the shape of the 3D microwells and tethering pillars for support. Additionally, specific ECM cues were provided in the 3D microenvironment via a degradable PEG‐ECM hydrogel for encapsulating the 3D microwells. There have been numerous 3D organoid platforms either modulating biomechanical forces or ECM composition independently, but never together. Especially, in the field of liver organoid technology, to the best of our knowledge, this is a unique platform developed with defined 3D biomechanical and ECM cues. Although not evaluated here, the stiffness of both the PEG microwells and encapsulated degradable PEG hydrogel can also be modulated by varying the PEG monomer type and concentration in the hydrogel. This could be especially crucial in studying diseases such as liver fibrosis, where the stiffness of the native tissue drives the disease phenotype.

We also found that controlling the 3D shape precisely was crucial to obtaining consistent cellular behavior between biological replicates. Moreover, tissue patterning and bifurcation of fate are achieved when precise mechanical forces are balanced in a multicellular structure, as demonstrated in the circular microarrays and 3D microwells. Patterned differentiation of hiHepatoblasts is not achieved when the tissue is just a spheroid of varying size and not tethered to any pillar. This was also demonstrated in our previous investigation of tissue patterning in 3D microtissues using the embryonic mouse progenitor cells.^[^ ^27^ ^]^ In addition to the shape, the encapsulation gel and its composition affected the bipotential differentiation. Specific ECM combinations promoted higher CK19 and HNF4a expression, whereas the noECM control had very low levels. Interestingly, as with microarray results, the Collagen 1+Fibronectin condition also had lower levels of HNF4a and CK19 expression. Future work involves investigating these findings further in terms of evaluating the identity of these cells. We will also evaluate if Collagen 1 + Fibronectin promotes higher proliferation or a stem cell maintenance state.

This study utilized the K3 iPSC line to establish our integrated 2D/3D platform approach for systematically investigating microenvironmental effects on hepatic differentiation. The robust and reproducible differentiation patterns observed across our 60 microenvironmental conditions, with consistent bipotential marker expression and spatial patterning, demonstrate the reliability and sensitivity of our platform for detecting microenvironmental effects. This foundational work establishes a process that can now be expanded to multiple iPSC lines at lower passages with comprehensive karyotypic characterization, enabling patient‐specific disease modeling and validation of findings across different genetic backgrounds.^[^ ^16^, ^17^, ^18^ ^]^ The approach demonstrated here establishes the platform's capability to detect nuanced microenvironmental responses, setting the stage for future investigations of inter‐line variability and precision medicine applications in liver disease modeling. In conclusion, this integrated platform provides a powerful framework for systematically dissecting the combinatorial effects of mechanical and biochemical cues on human hepatic development, representing an advancement toward more physiologically relevant in vitro models that can bridge the gap between high‐throughput screening and complex organoid systems.

## 4 Experimental Section

### hiPSC Cell Culture

The iPSCs were graciously received from the Duncan laboratory and were cultured according to the protocols established here.^[^ ^32^ ^]^ Briefly, iPSCs between passages 94–95 were thawed and cultured on geltrex (Invitrogen, #A1413301) coated 10 cm non‐TC treated cell culture dishes (Corning, #430 591). The geltrex coating was performed by using 0.05 mg mL^−1^ solution of geltrex in DMEM/F12 (Invitrogen, #11 330) that was left to coat the surface for an hour at 37 °C. The iPSCs were thawed and cultured in 8 mL of mTESR with 10 µm ROCK Inhibitor (Sigma‐Aldrich, #Y0503‐1 mg) and 40 ng mL^−1^ bFGF (Stemcell Technologies, #78 003). The media composition day 2 onward was mTESR supplemented with 40 ng mL^−1^ bFGF with daily media changes. For expanding the iPSCs, they were passaged at 50–60% confluency, and for differentiation experiments they were passaged at 60–70% confluency. The passage was performed by treating the cells with Versene 0.2 mg mL^−1^ EDTA) (Lonza, #17‐711E) for 3 min followed inactivation using media. The cells were then detached from the surface using gentle pipetting and centrifuged. A 1:3 passage was performed for expansion and seeded at 100% confluency (1 10 cm dish to 2–3 wells of 6 well plate) for differentiation experiments. For differentiation to hiHepatoblasts, the protocol established here^[^ ^32^ ^]^ was utilized. Briefly, RPMI 1640 (Invitrogen, #22 400), supplemented with 1% Pen/Strep (Millipore, #TMS‐AB2‐C) and 1% Non‐Essential Amino Acid (Millipore, #TMS‐001‐C) – referred to as RPMI media, was used with varying growth factor combinations. For differentiation Day 1 and 2, RPMI media was supplemented with 2% B27 (without Insulin) (Invitrogen, #0050129SA), 100 ng mL^−1^ Activin A (R&D systems, #338‐AC‐010), 10 ng mL^−1^ BMP4 (R&D, #314BP), and 20 ng mL^−1^ bFGF (Stemcell Technologies, #78 003) with daily media changes. For differentiation Day 3 – 5, RPMI media was supplemented with 2% B27 (without Insulin) and 100 ng mL^−1^ Activin A with daily media changes. For differentiation Day 6 – 10, RPMI media was supplemented with 2% B27 (with Insulin) (Invitrogen, #17 504 044), 20 ng mL^−1^ BMP4, and 10 ng mL^−1^ FGF2 with daily media changes. On Day 10, cells were expected to be in the hepatoblast differentiation stage and were harvested for microarray and microwell studies. From Day 11 onward, RPMI media supplemented with 2% B27 was used as the base media while growth factors were varied. Differentiation occurred at 37 °C, 5% CO_2_, and 21% oxygen at all stages.

### Microarray Fabrication and Culture

Polyacrylamide (PA) hydrogels were prepared following previous protocols.^[^ ^50^, ^51^, ^52^ ^]^ Briefly, 12 mm glass coverslips were etched by immersing them in 0.2 N NaOH (Sigma‐Aldrich 415413‐1L) for 1 h on an orbital shaker and then rinsing with dH_2_O. The coverslips were then air‐dried and placed on a hot plate at 110 °C until dry. For silanization, the cleaned coverslips were immersed in 2% v/v 3‐(trimethoxysilyl)propyl methacrylate (Sigma Aldrich 440159–500ML) in ethanol and placed on the shaker for 30 min, followed by a wash in ethanol for 5 min. The silanized coverslips were air‐dried and again placed on the hot plate at 110 °C until dry. For the fabrication of hydrogels with specific elastic moduli, prepolymer solution in dH20 with 8% acrylamide (Sigma‐Aldrich A3553‐100G) and 0.55% bis‐acrylamide (Sigma‐Aldrich M7279‐25G) was prepared to achieve elastic moduli of 25 kPa. The prepolymer solution was then mixed with Irgacure 2959 (BASF, Corp.) solution (20% w/v in methanol) at a final volumetric ratio of 9:1 (prepolymer:Irgacure). This working solution was then deposited onto Rain‐X (Amazon Rain‐X 800 002 245) coated slides (20 uL per coverslip) and covered with silanized coverslips. The sandwiched working solution was transferred to a UV oven and exposed to 365 nm UV A for 10 min (240E3 µJ). The coverslips with the hydrogels attached to them were immersed in dH_2_O at room temperature for a day in order to remove excess reagents from the hydrogel substrates. Before microarray fabrication, hydrogel substrates were thoroughly dehydrated on a hot plate for ≥15 min at 50 °C. Microarrays were fabricated as described previously, and detailed methods and images for microcontact printing are shown by Berg and Underhill.^[^ ^53^, ^54^, ^55^, ^56^ ^]^ ECMs (Table S1, Supporting Information) for arraying were diluted in 2×ECM printing buffer. To a final concentration of 250 µg mL^−1^ and loaded in a 384‐well V‐bottom microplate. To prepare 2× ECM protein printing buffer, 164 mg of sodium acetate and 37.2 mg of ethylenediaminetetraacetic acid (EDTA) were added to 6 mL dH2O. After solubilization, 50 µL of pre‐warmed Triton X‐100 and 4 mL of glycerol were added. 40 – 80 µL of glacial acetic acid was added, titrating to adjust the pH 4.8. A robotic benchtop microarrayer (OmniGrid Micro, Digilab) loaded with SMPC Stealth microarray pins (ArrayIt) was used to microprint ECM combinations from the 384 microwell plate to polyacrylamide hydrogel substrate, resulting in ≈600 µm diameter arrayed domains. Video S1 (Supporting Information) shows the deposition of ECM onto the polyacrylamide hydrogels. Fabricated arrays were stored at room temperature and 65% RH overnight and left to dry under ambient conditions in the dark. For the microarray seeding, cells harvested from a 100% confluent well of a 6‐well plate were seeded on 6 12 mm coverslips. The seeded microarrays were shaken gently every 20 min for 2 h after seeding. Media was refreshed after 4 h, and soluble factor treatment with HGF (20 ng mL^−1^), EGF (50 ng mL^−1^), or HGF & EGF was started on Day 11 with concentrations based on previous liver progenitor cell differentiation protocols.^[^ ^32^, ^46^ ^]^

### Microwell Fabrication and Culture

The protocol to prepare the wafer and PDMS molds for the microwells was established here. Using the PDMS models, PEG hydrogel substrates were prepared by adapting previous protocols.^[^ ^27^, ^52^, ^57^ ^]^ A total of 12 mm circular coverslips were immersed in 0.1 N NaOH for 1 h, rinsed with DiH_2_O and placed on a hot plate at 110 °C until dry. The NaOH‐treated coverslips were activated by immersion in 2% (v/v) 3‐(trimethoxysilyl)propyl methacrylate in ethanol and placed on a shaker for 30 min. The activated coverslips were immersed in ethanol on the shaker for 5 min and again dried on a hot plate at 110 °C. A 111.11 mg mL^−1^ 10 kDa 4‐Arm PEG‐acylate (Laysan Bio) prepolymer solution was prepared in 1× PBS. This solution was mixed with Irgacure 2959 (BASF) solution (100 mg mL^−1^ in methanol) at a volumetric ratio of 9:1 (prepolymer to Irgacure) to achieve a final PEG concentration of 100 mg mL^−1^. The solution was then degassed under vacuum. For PEG molding, the 1 mm gasket was positioned around the feature array to be used, leaving ≈1 mm between the raised platform and the gasket. A 50 µL droplet of prepolymer solution was deposited on the mold. A pipette tip was used to spread the prepolymer solution across the mold and knock bubbles from the mold features. The droplet was covered with an acrylated coverslip, which was lightly pressed against the gasket. The assembly was exposed to UV light using a Spot UV Curing System (OmniCure S1500, Excelitas Technologies) with a 320–390 nm Filter and adjustable collimating adapter, at an intensity of ≈50 mJ cm^−2^ for 30–60 s. The coverslip was then carefully removed from the mold and immersed in PBS in a 24‐well plate. Video S2 shows the fabrication of a PEG microwell using a PDMS mold with various geometries. Prior to use in culture, the substrates were sterilized by immersion in PBS supplemented with 1% Pen/Strep under UVC for 30 min. For the microwells, cells harvested from a 100% confluent well of a 6‐well plate were seeded into 3 microwells, each on a separate 12 mm coverslip. The media was refreshed after 4 h, and soluble factor treatment was started on Day 11.

### Immunohistochemistry

Microarray samples were fixed in 4% w/v paraformaldehyde, 1x PBS for 15 min. Fixed samples were then permeabilized with a 0.25% v/v Triton X‐100, 1× PBS for 10 min and incubated in 1% w/v BSA and 0.25% v/v Triton X‐100, 1× PBS for one hour at room temperature. After blocking, samples were incubated overnight at 4 °C with primary antibodies in blocking buffer. Samples were subsequently washed with 1x PBS thrice and then incubated for one hour at room temperature with secondary antibodies diluted in blocking buffer (Table S1, Supporting Information). Finally, samples were mounted in Fluoromount G with DAPI (Southern Biotech, 0100–20).

Microwells were fixed by immersion in paraformaldehyde (4% [v/v] in 1× PBS) for 30 min at room temperature. Fixed samples were permeabilized by immersion in Triton ×‐100 (0.5% [v/v] in 1× PBS) for 1 h at room temperature. Samples were incubated in blocking buffer (1% [v/v] BSA in 1× PBS) for 1 h at room temperature. Primary antibody solutions were prepared by diluting one or more of the following antibodies in blocking buffer: mouse anti‐HNF4a (1/200 from stock, Abcam ab41898), goat anti‐CK19 (1/200 from stock, Abcam ab52625). A 50 µL droplet of primary antibody solution was added to the recessed loading well of each substrate. Samples were incubated overnight at room temperature on a shaker. Samples were rinsed via 3 × 15‐min washes in PBS on a shaker. Secondary antibody solutions were prepared by diluting one or more of the following secondary antibodies in blocking buffer: DyLight 550‐conjugated donkey anti‐mouse IgG (1/50 from stock, Abcam, ab98767) and DyLight 488‐conjugated donkey anti‐goat IgG (1/50 from stock, Abcam, ab96935). A 50 µL droplet of secondary antibody solution was added to the recessed loading well of each substrate. Samples were incubated overnight at room temperature on a shaker. Samples were rinsed via 2 × 15‐min washes in PBS on a shaker. Samples were incubated in DAPI solution (Invitrogen D1306) for 1 h, and briefly rinsed in PBS. A droplet of liquid mountant (ProLong Diamond Antifade, Invitrogen) was added to each substrate, and the coverslips were mounted onto standard microscope slides. Mounted samples were cured for at least 24 h at room temperature. Once cured, samples were sealed with clear nail polish.

### Imaging and Analysis

The microarrays for the immunostaining were imaged using an Axioscan.Z1 Slide Scanner and 10X objective. A wide tile region was defined for the whole array region, which was then stitched offline using Zen and exported into TIFF Images for each individual channel. Images of entire arrays were converted to individual 8‐bit TIFF files per channel (i.e., red, green, blue) by Fiji (ImageJ version 1.52p).^[^ ^58^ ^]^ The images were cropped in MATLAB (version R2018b) to separate each array into a single image. Positional information for each array was automatically calculated using its relative position from the positional dextran‐rhodamine markers. CellProfiler (version 4.0.0)^[^ ^59^ ^]^ was used to get per‐cell measurements for each channel. Nuclei were identified using the DAPI channel image using the IdentifyPrimaryObject module, and other stains were associated with a specific nucleus by looking at the red/green stain around these nuclei using the IdentifySecondaryObject module. The MeasureObjectIntensity module was used to quantify single‐cell intensity. The data were exported to CSV files that were then imported into RStudio for data visualization.

The fluorescently stained microwells were imaged using a Zeiss Confocal Microscope (LSM 880). The z‐stacks obtained were rendered in Imaris 9.7 for 3D segmentation of cells. The surfaces were identified in each different channel corresponding to a specific secondary antibody using Imaris. The data was transferred to RStudio for data visualization.

### Data Processing and Statistics

All microarray and microwell experiments consisted of at least three biological replicates, with 15 technical replicates, or islands or a microwell, per biological replicate per combination of treatment and readout. For comparison between conditions in this study, Wilcoxon tests were performed using the Wilcox test function in R. *p*‐Values of < 0.05 were considered significant. For the line graph demonstrating quantification of a readout as a function of the radius, a 95% confidence interval was calculated and displayed using the geom_smooth function in R. PCA Analysis was done in R using FactoMineR and factoextra. Input variables were normalized, and the contribution of each variable to the principal components was analyzed. Briefly, contributions of specific ECM and growth factor contributions (qualitative variables) were plotted on the principal component planes. Furthermore, relationships between principal components and output metrics, such as the differentiation markers (quantitative variables) were shown using loading vectors, to interpret the positions of the ECM and growth factors to the output metrics.

## Conflict of Interest

The authors declare no conflict of interest.

## Supporting information



Supporting Information

Supplemental Video 1

Supplemental Video 2

## Data Availability

The data that support the findings of this study are available from the corresponding author upon reasonable request.
