# Supplementary material for: Effect of 2D and 3D ECM and Biomechanical Cues on Human iPSC‐Derived Liver Progenitor Cell Differentiation
Source: Adv Healthc Mater. 2025 Sep 29;15(4):e01370. doi: 10.1002/adhm.202501370 (PMC12836466; doi:10.1002/adhm.202501370)
Supplement: Supplementary file 1 — Supporting Information [file ADHM-15-0-s003.docx]

**Supporting Information**

**Effect of 2D and 3D ECM and Biomechanical Cues on Human iPSC-derived Liver Progenitor Cell Differentiation**

Ishita Jain, Brock Grenci, Hyeon Ryoo, Yang Yuan, Salman R. Khetani, Gregory H. Underhill

**Supplementary Video Captions**

**Video S1.** Microcontact printing procedure for array fabrication

**Video S2.** Fabrication of 4-arm PEG acrylate microwells from PDMS molds
